# The value of a simple method to decrease diagnostic errors in Turner syndrome: a case report

**DOI:** 10.1186/s13256-021-02673-0

**Published:** 2021-02-18

**Authors:** Seyedetahere Mousavi, Batool Amiri, Saidee Beigi, Mohammadreza Farzaneh

**Affiliations:** 1 Pediatric Endocrinology, School of Medicine, Bushehr University of Medical Sciences, Bushehr, Iran; 2 Clinical Research Development Center, Bushehr University of Medical Sciences, Bushehr, Iran; 3 Royal Australian College of General Practitioners, The Melanoma Centre, Brisbane, Australia; 4 Molecular Pathology and Cytogenetic, School of Medicine, Bushehr University of Medical Sciences, Bushehr, Iran

**Keywords:** Short stature, Turner syndrome, Karyotype, Failure to thrive

## Abstract

**Introduction:**

Turner syndrome is a genetic disorder in females and is the result of complete or partial loss of an X chromosome during fertilization. The missing X chromosome is originally either from the mother's ovum or the father's sperm cell. Approximately 45% of patients have the 45,X karyotype and the rest have other variants of Turner syndrome, which are either mosaicism patterns or structural abnormalities of the X chromosome. Here, we report a case of Turner syndrome that is the fifth case of Turner syndrome with balanced Robertsonian translocation of (13;14)(q10;q10), and the sixth case with 44,X chromosomes, reported in the literature thus far.

**Case presentation:**

A 10.3-year-old Persian girl was brought to our clinic by her parents, with the complaint of failure to thrive and short height. She had been examined and investigated by endocrinologists since the age of 4 years, but no definite diagnosis was made. At the time of presentation, she had been through three provocative growth hormone tests and had been on no medications for about a year. Her physical examination revealed mild retrognathia and micrognathia. Initially, she was started on somatropin treatment which, after 12 months, did not appropriately improve her height velocity. Therefore, a more thorough physical examination was performed, in which high arched palate and low posterior hairline were observed. There was also a difference between target height and patient height standard deviation scores. Karyotype study was requested, and Turner syndrome was confirmed.

**Conclusion:**

The diagnosis of this case was not straightforward, both because the somatic presentations were not obvious, and because the physicians had not looked for them when performing the physical examinations. This case report introduces a rare 44,X chromosome karyotype of Turner syndrome and highlights the value in using the difference between target height and patient height standard deviation scores as a simple and inexpensive tool for diagnosis of this syndrome.

## Introduction

Turner syndrome (TS) is a genetic disorder in females that is the result of complete or partial loss of an X chromosome during fertilization. It occurs in approximately one in 2500 female live births. The missing X chromosome is originally either from the mother's ovum or the father's sperm cell. Approximately 45% of patients have the 45,X karyotype [1–3] and the rest have other variants of TS, which are either mosaicism patterns or structural abnormalities of the X chromosome.

TS has a wide range of clinical symptoms. The most common symptoms are short stature and primary gonadal deficiency [1–3]. The patient may have somatic features such as high arched palate, low posterior hairline, and cervical web [1]. Other clinical features include horseshoe kidney, coarctation of the aorta, diabetes, hypothyroidism, hypertension, hearing loss, osteoporosis, bone fracture, and gastrointestinal problems. Skeletal anomalies such as cubitus valgus, Madelung deformity, and short fourth and fifth metacarpal and metatarsal bones are also seen [1–3]. Diagnosis of TS is based on suggestive clinical findings and karyotype analysis of peripheral blood, which reveals the numerical and/or structural abnormalities of the X chromosome.

This report presents a rare karyotype of TS. The patient’s diagnosis was not made until she was 10 years of age, as her clinical signs and symptoms were not obvious enough to be observed by endocrinologists. The delay in TS diagnosis could have been avoided if it had been considered as one of the differential diagnoses of short stature in girls with subtle signs on physical examination. The difference between patient height standard deviation score (SDS) and target height SDS (TH SDS) is also a simple tool which can be used in the evaluation of short-stature patients, and was a valuable guide in this rare case, allowing us to proceed with further evaluations.

## Case presentation

A 10.3-year-old girl presented to the Children’s Walk-in Endocrinology Center (CWEC) in Bushehr, located on the northern shore of the Persian Gulf, accompanied by her parents, with the chief complaint of short stature and failure to thrive. She was the first child of Persian non-consanguineous parents from southern Iran. Her father was an engineer and her mother was a teacher. The family was of high socioeconomic and normal psychosocial status. The second child of the family was a 4.5-year-old girl with normal growth and development who had not required any specific medical attention. The patient was the product of a normal vaginal delivery at a gestational age of 32 weeks, with a birth weight of 2400 g. Her birth length and head circumference were 41 and 30 cm, respectively. She was conceived when her mother was 26 and her father 32 years old. According to our patient's infantile weight and height growth chart, she was in the third percentile in the first 4 months, and continued to ascend in the third percentile for age and sex with normal height velocity up to the age of 2 years. Sufficient information about her growth velocity was not available beyond that age.

She had been examined intermittently by endocrinologists between the ages of 4 and 9 years due to failure to thrive and short stature. Three provocative growth hormone (GH) tests were performed during that time. The first was conducted with levodopa provocation when she was 4.9 years old. The second and third tests were carried out with clonidine provocation (5 mcg/kg) when she was 7.8 and 9.1 years old, respectively. The results of GH provocation tests were 5.11 ng/mL (at the age of 4.9 years), 10.5 ng/mL (at the age of 7.8 years), and 6.99 ng/mL (at the age of 9.1 years). Two of the tests showed no response, and therefore GH replacement therapy [somatropin, NordiLet pen 5 mg/1.5 mL (35 mcg/kg/day)] was initiated and delivered via subcutaneous injections, nightly, six times per week. Eight months later, the treatment was ceased because the patient had not gained acceptable growth velocity and height. In addition to the above treatment, she was on oral supplements including zinc 5 mg daily and multivitamins, but her parents were not aware of the exact dosage and type of vitamins. Other pathologies which were initially checked in this case were thyroid function tests, screening for celiac disease, and kidney and liver function tests, which were all at normal levels (Table 1). Plain X-ray of her left hand and wrist did not detect any abnormalities, and the bone age, according to Greulich and Pyle, was 7 years for a chronological age of 8.2 years. There was no other detailed information about her growth velocity and management.

**Table 1:** Laboratory findings before presenting to Children’s Walk-in Endocrinology Center

Test (normal range)	Age 4.9 years	Age 7.8 years
WBC (4500–15,000 cells/µL)	8900 cells/µL	9100 cells/µL
RBC (4–5.4 × 10^6^/µL)	4.49 × 10^6^/µL	5.16 × 10^6^/µL
Hemoglobin (> 11.5 g/dL)	12.6 g/dL	13.6 g/dL
Mean corpuscular volume (> 77 fL)	81.7 fL	75.6 fL
Platelet count (150,000–450,000 cells/µL)	292,000 cells/µL	337,000 cells/µL
TSH (0.6–5.5 µIU/mL)	–	1.4 µIU/mL
Thyroxine (6–13.8 µg/dL)	–	8.3 µg/dL
BS (55–140 mg/dL)	–	84 mg/dL
TTG IgA antibody (< 12 IU/L)	0.7 IU/L	–

*WBC* white blood cells, *RBC* red blood cells, *TSH* thyroid-stimulating hormone, *BS* blood sugar, *TTG* tissue transglutaminase, *IgA* immunoglobulin A

By the time the patient presented to CWEC, she had not been taking any prescribed or over-the-counter medications for about a year. She was a fifth grade student and a bit behind her class. She was short and slim, with height of 118 cm. Height standard deviation score (SDS) was −3.3 and weight was 19 kg. Her mother's height was 159.5 cm and father's height was 167cm.

Mid-parental height (MPH) = (mother’s height + father’s height/2) − 6.5 cm = 156.75 cm

Target height (TH) = 161.25 cm (calculated by Tanner’s method with an additional correction for secular trend = MPH + 4.5 cm) [4].

TH SDS was −0.3. The difference between TH SDS and patient height SDS was –3 SD.

On the first consultation, the vital signs were normal (blood pressure 90/60 mmHg, heart rate 98 beats per minute, and temperature 36.8 °C). Physical examination indicated mild retrognathia and micrognathia. The parents claimed that the face was similar to that of her paternal grandmother. She had no signs of puberty. Neurological evaluation and other aspects of physical examination including chest, heart, abdomen, extremities, and genitalia were normal.

Initially, we took into account her being small for gestational age (SGA) at birth (low birth weight and height SDS < −1.88 after the age of 2 years [4]), being unable to reach the normal growth chart since the age of 4 years, and having had two unresponsive provocative GH tests, and diagnosed her with SGA without catch-up growth. She was started on GH [somatropin (NordiLet pen 5 mg/1.5 mL) 35 mcg/kg/day, subcutaneous injections seven nights per week]. She was also put on a regimen of multivitamins (Nature Made Multivitamin Complete tablet), one tablet six times per week. Kidney, liver, and thyroid function, complete blood count, biochemistry, urinalysis, and celiac disease studies were again requested and were all with normal ranges (Table 2). One year after being on this treatment cocktail, the height velocity had not improved according to our expectations. Height SDS was −3.4, and increment in height SDS was −0.1.

**Table 2:** Findings of laboratory tests performed at Children’s Walk-in Endocrinology Center

Test (normal range)	Age: 10.3 years	Age: 12.5 years	Age: 14 years
CBC			
WBC (4500–15,000/µL)	7470/µL	7380/µL	7700/µL
RBC (4–5.4 × 10^6^/µL)	5.24 × 10^6^/µL	4.97 × 10^6^/µL	4.74 × 10^6^/µL
Hb (> 11.5 g/dL)	13.9 g/dL	14.1 g/dL	13.7 g/dL
MCV (> 77 fL)	79.8 fL	80.7 fL	82.7 fL
Platelet count (150,000–450,000 cells/µL)	214,000 cells/µL	151,000 cells/µL	195,000 cells/µL
BUN (6–24 mg/dL)	8.2 mg/dL		
Creatinine (0.3–0.7 mg/dL)	0.4 mg/dL		
Sodium (135–145 mEq/L)	138 mEq/L		
Potassium (3.5–5.5 mEq/L)	3.9 mEq/L		
BS (55–140 mg/dL)	84 mg/dL	86 mg/dL	87 mg/dL
AST (< 35 IU/L)	26 IU/L	23 IU/L	
ALT (< 35 IU/L)	20 IU/L	18 IU/L	
TSH (0.6–5.5 µIU/mL)	3.29 µIU/mL	2.93 µIU/mL	3 µIU/mL
Total thyroxine (6–13.8 µg/dL)	8.62 µg/dL	6 µg/dL	8.5 µg/dL
Calcium (8.8–10.5 mg/dL)	9.6 mg/dL		
Phosphor (3.5–6.4 mg/dL)	5.9 mg/dL		
Anti-tissue transglutaminase IgA (< 12 U/mL)	9.2 U/mL	2.5 U/mL	
Alkaline phosphatase (180–1200 IU/L)	402 IU/L		
ACTH (7.2–63.6 pg/mL)	52.28 pg/mL		
Cortisol (171–536 nmol/L)	978.9 nmol/L		
IGF-1 (87.4–399 ng/mL)	182.49 ng/mL	680.4 ng/mL	302 ng/mL
Urinalysis	WBC = 0, RBC = 0, SG = 1.018		
Stool exam	WBC = 0, RBC = 0, Parasite = negative		

*CBC* complete blood count, *WBC* white blood cells, *RBC* red blood cells, *Hb* hemoglobin, *MCV* mean corpuscular volume, *BUN* blood urea nitrogen, *BS* blood sugar, *AST* aspartate aminotransferase, *ALT* alanine aminotransferase, *TSH* thyroid-stimulating hormone, *IgA* immunoglobulin A, *ACTH* adrenocorticotropic, *IGF-1* insulin-like growth factor 1, SG specific gravity

At this time, we performed another physical examination and found that the patient had a high arched palate and low posterior hairline. She had no other somatic signs of TS including short neck, scoliosis, cubitus valgus, short fourth metacarpal or metatarsal bone, or Madelung deformity. The next and final step of our investigation was to request karyotype screening, which confirmed a diagnosis of TS, even though her karyotype was not typical of TS.

**Fig. 1:**
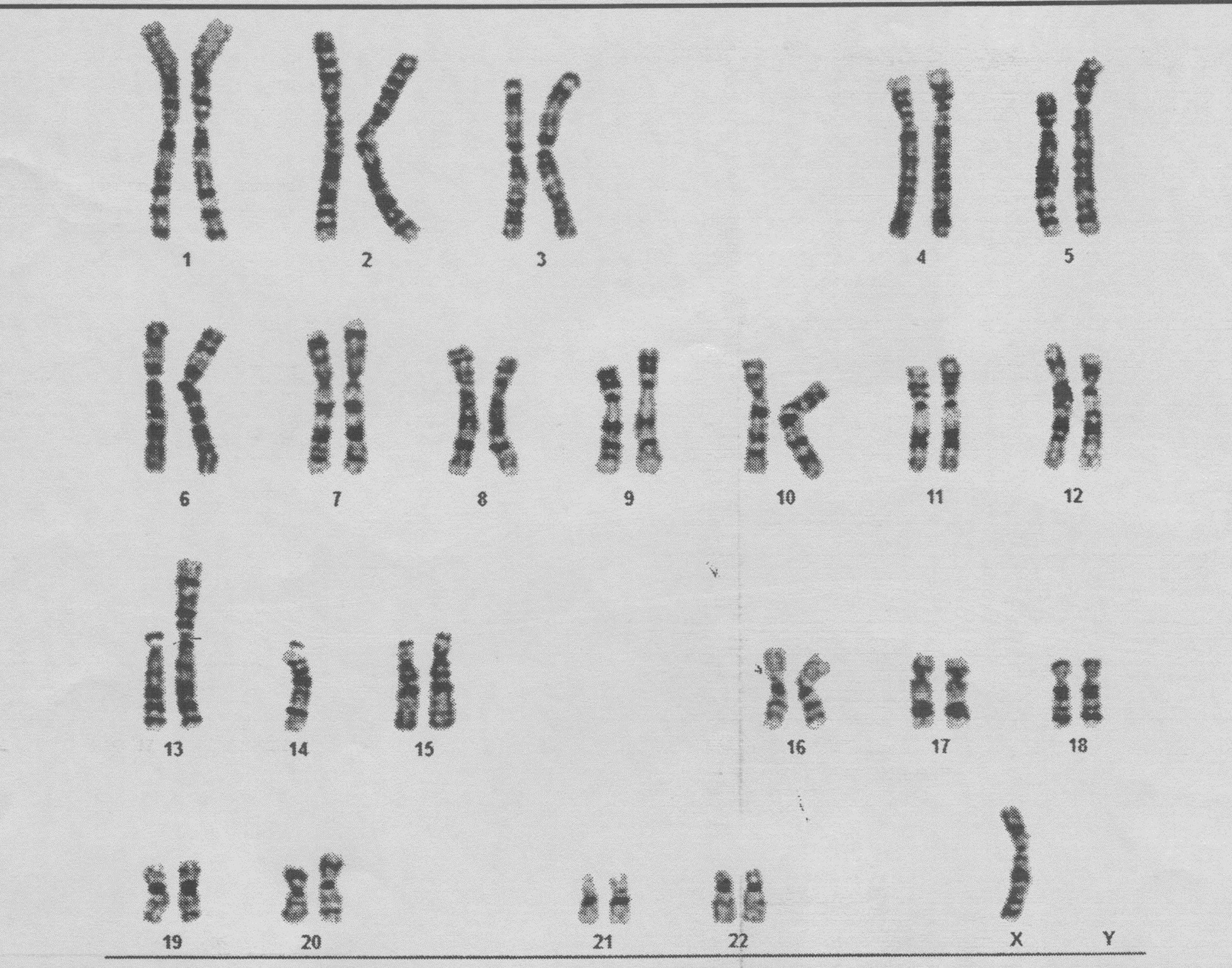
Karyotype of the patient which shows Turner syndrome with 44X,der(13;14)(q10; q10)

Twenty-one metaphase spreads were studied based on the trypsin-Giemsa G-band (GTG) technique at 400–550-band resolution. Chromosome analysis revealed an abnormal female chromosome complement in all cells examined with a single X chromosome and a balanced Robertsonian translocation between the long arms of chromosomes 13 and 14. The result showed TS with 44,X,der(13;14)(q10;q10) karyotype (Fig. 1).

**Fig. 2:**
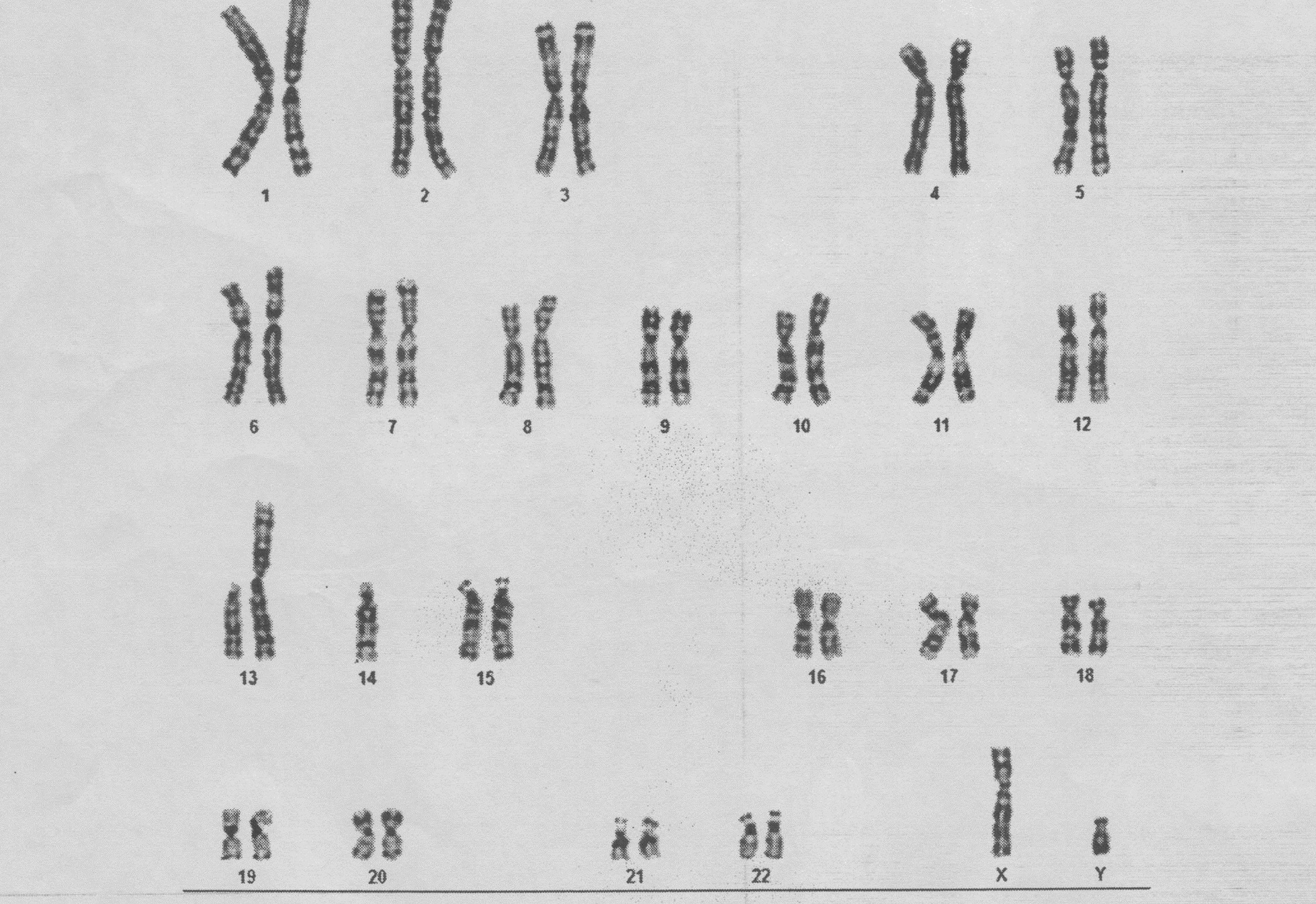
Karyotype of the father: balanced Robertsonian translocation between the long arms of chromosomes 13 and 14 (45,XY,der (13;14)(q10; q10)

Karyotype studies of the parents and the only sibling (the patient's 6-year-old sister) were requested. Karyotype analysis of the mother and sister were normal (46,XX), but the father's analysis showed a balanced Robertsonian translocation between the long arms of chromosomes 13 and 14, similar to the patient’s [45,XY,der(13;14)(q10;q10)] (Fig. 2). It appeared that the balanced translocation was transmitted from the father. We did not determine whether the origin of the single X chromosome was paternal or maternal.

When the TS diagnosis was confirmed at the age of 11.9 years, we increased the dose of GH to 50 mcg/kg/day, and oral oxandrolone (0.04 mg/kg/nightly) was added to the treatment. Cardiac and renal evaluations were normal. Her annual blood pressure measurements were within the normal range (on average 85/60 mmHg). Abdominal sonography demonstrated a rudimentary uterus, and ovaries were not visible. The patient was reviewed every 6 to 12 months, and her height, weight, and growth velocity were measured, in addition to general physical examination and assessment of the recent blood results. In the first 15 months of her treatment and follow-up, her height increased 6 cm (Table 3).

**Table 3:** Height of the patient at the time of the diagnosis of Turner syndrome and follow-ups

Patient age (years)	Height (cm)	Height SDS	Delta height SDS
11.75	124	−3.4	
12.4	127.5	−3.5	
13	130	−3.9	−0.5 SD
13.5	133.5	−3.8	
14	137	−3.6	+0.3 SD

The increase in height SDS was −0.5 SD, which was not sufficient. Once again, we checked thyroid function and serum anti-tissue transglutaminase (TTG) immunoglobulin A (IgA), and performed fecal tests to detect any evidence of autoimmune thyroid, celiac, or inflammatory bowel diseases. All the results were within the normal range (Table 2). By then, she was 14 years old, with height of 137 cm (SDS = −3.6) and weight of 31 kg. The height increment in the second year of treatment was +0.3 SD (Table 3).

This young girl is still on GH (NordiLet pen 50 mcg/kg/day) and oxandrolone treatment. We have not yet started her on estrogen replacement therapy, as the ultimate height increase was the first concern, but it will be considered in our next step of treatment. The last bone age measurement, according to Greulich and Pyle, was 11.9 years, with chronological age of 13.5 years.

If the diagnosis had been made earlier and the GH had started with a higher dose from the beginning, her height would be predicted to be taller [5].

### Patient perspective

When the condition was discussed with her parents, they became anxious and were in denial at first. Later on, they accepted the situation, especially when the syndrome characteristics were explained to them in full detail. They were very cooperative, and encouraged their daughter to attentively take the medications. However, the parents decided to not inform the patient of the exact diagnosis, as they were worried it might affect her mental health. Although both the patient and her parents were unhappy and disappointed that it took more than a decade for the diagnosis to be clarified, despite the fact that they were frequently seeking medical attention, they acknowledged that TS is not a common condition, and were there higher awareness of the symptoms and diagnosis of this syndrome among physicians, particularly endocrinologists, it could have been managed earlier and the outcome would be more acceptable.

## Discussion

Our patient is the fifth case of TS reported in the literature with balanced Robertsonian translocation of (13;14)(q10;q10), and the sixth case with 44,X chromosomes. In her first presentation to our center, at the age of 10 years, the patient was a severely short girl with some somatic signs of TS, which unfortunately had not been diagnosed until then. In this scenario, the limited knowledge of medical practitioners of possible etiologies of failure to thrive and short stature in young girls was the main reason for the delayed diagnosis. Therefore, this highlights the importance of expanding our knowledge of differential diagnoses and paying attention to the difference between patient height standard deviation score (SDS) and target height SDS, and any subtle sign on physical examination. In addition to the above, we also recommend considering TS in every short-stature girl with a diagnosis of SGA without catch-up growth or growth hormone deficiency (GHD) who has not reached normal growth chart height with GH treatment.

Torun *et al*. emphasized that TS should be considered in every short girl as the etiology of short stature. Even if no phenotypic signs consistent with TS are detected, karyotype analyses should be carried out [6]. In our case, if therapy had been initiated before the patient had deviated significantly from the normal growth curve, she would have had a greater potential for catch-up growth, and greater height potential would have remained. During the initial consultations, diagnosis of TS was ignored for the following reasons:

1. The patient was born with low birth weight and had not gained normal growth after the age of 4 years.
2. She was frequently managed by two senior endocrinologists, whose opinion and diagnosis were relied on by other physicians.
3. She had two unresponsive GH provocative tests which, in addition to her severe short stature, made the likelihood of GHD very high.

Clinical signs in favor of TS in our case are the height SDS greater than two standard deviations below TH SDS [7] and some somatic signs (high arched palate, posterior hairline), but it was the karyotyping tests which confirmed the diagnosis of TS. Although the majority of TS cases have 45X or mosaicism variant karyotypes, our case has 44 chromosomes and also a balanced translocation of a pair of somatic chromosomes (13;14).

There have been only four case reports of TS with 44 chromosomes and this type of translocation in the literature. The first case was reported in 1985 by Salamanca. The patient had 44,X,der(13;14) translocation, the same as our patient. The (13;14) translocation was maternally inherited but the study did not investigate the paternal origin of X chromosome non-disjunction [8].

The second was reported by Laszlo *et al*., which was a case of 44,X streak gonad syndrome with balanced Robertsonian translocation of (13;14) whose mother carried the translocation [9].

The third was a case of TS reported by Krajinovic *et al*. in 1994. The patient was a 16-year-old girl with short stature, amenorrhea, and phenotypic characteristics of TS. The karyotype was 44,X,*t*(13q;14q). Similar to our case, the translocation was inherited from her father. Her mother and sister had normal female karyotypes. The X chromosome was also paternally inherited [10].

The fourth case was reported by da Silva *et al*. in 2006. The patient was a 5-year-old girl with short stature and facial dysmorphia. Chromosome analysis showed X monosomy and balanced Robertsonian translocation between chromosomes 13 and 14 [44,X,der(13;14)(q10; q10)], which is similar to our patient. However, in da Silva’s case, the karyotype of the father was normal, and it was the mother who carried a balanced translocation. The origin of the patient's X chromosome was paternal, and chromosomes 13 and 14 showed biparental inheritance [11].

The last reported case of TS with 44 chromosomes was an 11-year-old girl with short stature and clinical signs of TS, reported by Mariño *et al*. Her balanced translocation was different from the other reported cases of TS and 44 chromosomes. This patient’s karyotype showed X monosomy and Robertsonian translocation between chromosomes 15 and 22 [44,X,der(15;22)(q10; q10)]. Paternal karyotyping was not performed [12].

## Conclusion

Our case is the fifth case of TS with balanced Robertsonian translocation of (13;14)(q10;q10) reported in the literature, and the sixth reported case with 44,X chromosomes in patients with TS. Poor attention to the clinical symptoms of TS in a short girl and failing to consider the difference between patient height SDS and target height SDS resulted in a delay in the diagnosis of this case. It is crucial that physicians, and endocrinologists in particular, consider ruling out TS in every short girl even if they have no other somatic signs of TS, especially if the diagnosis is in doubt or the response to the usual treatment is not acceptable. This case also points out that the first examination should always be as thorough as possible, and physicians should not rely solely on the diagnosis and investigations previously performed. Furthermore, this case report illustrates the value in the measurement of the difference between target height SDS and patient height SDS as a simple and inexpensive tool for the diagnosis of TS, highlighting the rare karyotype forms.

## Data Availability

Data are available by request.

## Abbreviations

SDS: Standard deviation score
TS: Turner syndrome
GH: Growth hormone
TH SDS: Target height standard deviation score
CWEC: Children’s Walk-in Endocrinology Center
MPH: Mid-parental height
SGA: Small for gestational age
GTG: Trypsin-Giemsa G-band
GHD: Growth hormone deficiency
